# HLH-1 Modulates Muscle Proteostasis During *Caenorhabditis elegans* Larval Development

**DOI:** 10.3389/fcell.2022.920569

**Published:** 2022-06-06

**Authors:** Khairun Nisaa, Anat Ben-Zvi

**Affiliations:** Department of Life Sciences, Ben-Gurion University of the Negev, Beer Sheva, Israel

**Keywords:** *Caenorhabditis elegans* (c. elegans), development, MyoD, *hlh-1*, myosin, proteostasis, chaperone

## Abstract

Muscle proteostasis is shaped by the myogenic transcription factor MyoD which regulates the expression of chaperones during muscle differentiation. Whether MyoD can also modulate chaperone expression in terminally differentiated muscle cells remains open. Here we utilized a temperature-sensitive (ts) conditional knockdown nonsense mutation in MyoD ortholog in *C. elegans,* HLH-1*,* to ask whether MyoD plays a role in maintaining muscle proteostasis post myogenesis. We showed that *hlh-1* is expressed during larval development and that *hlh-1* knockdown at the first, second, or third larval stages resulted in severe defects in motility and muscle organization. Motility defects and myofilament organization were rescued when the clearance of *hlh-1(ts)* mRNA was inhibited, and *hlh-1* mRNA levels were restored. Moreover, *hlh-1* knockdown modulated the expression of chaperones with putative HLH-1 binding sites in their promoters, supporting HLH-1 role in muscle maintenance during larval development. Finally, mild disruption of *hlh-1* expression during development resulted in earlier dysregulation of muscle maintenance and function during adulthood. We propose that the differentiation transcription factor, HLH-1, contributes to muscle maintenance and regulates cell-specific chaperone expression post differentiation. HLH-1 may thus impact muscle proteostasis and potentially the onset and manifestation of sarcopenia.

## 1 Introduction

The ability to maintain a functional proteome throughout life is vital for long-term organismal health (Shai et al., 2014; Sala et al., 2017; Meller and Shalgi, 2021). Cells cope with protein damage by employing highly conserved quality control systems that repair or remove the damaged proteins to maintain proteostasis (Bar-Lavan et al., 2016a; Bett, 2016; Jackson and Hewitt, 2016). The cellular chaperone machinery is involved in many cellular processes, including *de novo* folding, assembly and disassembly of protein complexes, protein translocation across membranes, proteolytic degradation, and unfolding and reactivation of stress-denatured proteins (Makhnevych and Houry, 2012; Bar-Lavan et al., 2016a; Bett, 2016; Jackson and Hewitt, 2016; Craig, 2018; Nillegoda et al., 2018). Chaperones unfold, refold and reactivate proteins to gain or recover their function (Rosenzweig et al., 2019). The regulation and specificity of chaperone-based reactions can be mediated by co-chaperones choosing the substrate, presenting it to the chaperone, and coordinating chaperone-substrate binding and release cycles (Kampinga and Craig, 2010; Bar-Lavan et al., 2016a; Rosenzweig et al., 2019). As its folding advances, a substrate can be identified by different chaperones or co-chaperones, resulting in substrate overlap, shuffling, and collaboration between various chaperone machinery (Bar-Lavan et al., 2016a; Rosenzweig et al., 2019). These chaperone or co-chaperone interactions constitute the chaperone network (Brehme et al., 2014; Shemesh et al., 2021).

Most chaperones are ubiquitously expressed, yet chaperone and co-chaperone expression levels display tissue-specific patterns (Brehme et al., 2014; Shemesh et al., 2021). These patterns are conserved and linked to the cellular proteome’s folding demands, suggesting that the proteome diversity and differential cellular folding requirements shape the chaperone network in multicellular organisms (Shemesh et al., 2021). Such tailoring of the chaperone system to the proteome needs enables cells to respond to their unique folding requirements, contributing to tissue-specific vulnerability in protein-misfolding diseases (Basha et al., 2020; Thiruvalluvan et al., 2020; Vonk et al., 2020). While it is still an open question how chaperone expression is regulated in a tissue-specific manner, a role for differentiation transcription factors that establish the cell-specific proteome in defining the chaperone network is well-established (Nisaa and Ben-Zvi, 2021). One well-characterized example is the role of the myogenic transcription factor, MyoD, and its’ *Caenorhabditis elegans* ortholog, HLH-1 (Fukushige and Krause, 2005), in regulating chaperone expression during muscle differentiation (Sugiyama et al., 2000; Bar-Lavan et al., 2016b; Echeverria et al., 2016; Tiago et al., 2021).

The chaperone network is rewired during muscle differentiation, resulting in the induced expression of some chaperones and repression of others (Bar-Lavan et al., 2016b; Echeverria et al., 2016; Nisaa and Ben-Zvi, 2021). This muscle-specific expression pattern remains consistent across development and aging (Brehme et al., 2014; Shemesh et al., 2021). Chaperones that are upregulated during myogenesis also show muscle-specific differential expression in adult muscle tissues (muscle chaperones). This pattern is conserved from human to worm (Bar-Lavan et al., 2016b; Shemesh et al., 2021). MyoD/HLH-1 can regulate the expression of most muscle chaperones associated with this conserved muscle expression pattern (Bar-Lavan et al., 2016b; Echeverria et al., 2016), and it has functional binding sites at the promoters of most muscle chaperones (Sugiyama et al., 2000; Bar-Lavan et al., 2016b). Disrupting MyoD/HLH-1 function or mutating its’ binding sites abolish muscle chaperones’ expression during myoblasts differentiation, while its ectopic expression induces chaperone expression (Bar-Lavan et al., 2016b; Echeverria et al., 2016). While MyoD/HLH-1 modulates muscle chaperone expression during differentiation, a role for HLH-1 in maintaining muscle proteostasis later in life was not determined. Here, we asked whether HLH-1 plays a role in muscle maintenance post myogenesis by knocking down *hlh-1* expression at different points during *C. elegans* development.

## 2 Materials and Methods

### 2.1 Nematodes and Growth Conditions

**FIGURE 1:**
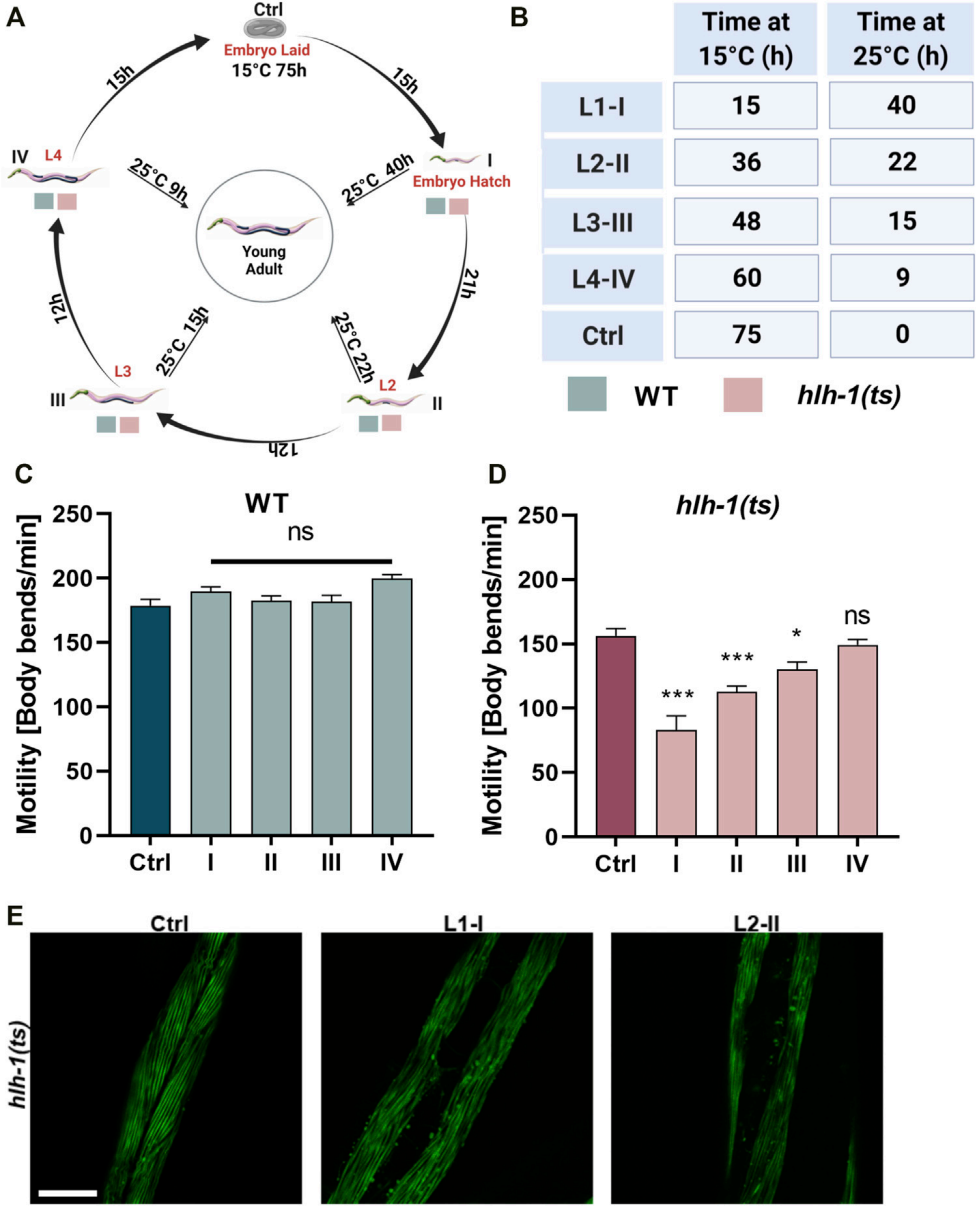
Differentiation transcription factor HLH-1 is required for muscle maintenance during larval development. **(A)** Schematic representation of the experimental setup. Wild type (WT) or *hlh-1(ts)* embryos were placed on seeded plates at 15°C. Animals were maintained at 15°C (Ctrl) or shifted to 25°C at the indicated times, corresponding to specific larval stages (L1-I, L2-II, L3-III, and L4-IV). Young adults (YA, before the onset of egg-laying) were then analyzed for motility, myofilament organization, and chaperone expression. **(B)** A list of treatments duration (in hours) at 15°C and 25°C for each group (Ctrl and I-IV). **(C–D)** Motility rates of temperature-shifted YA. Thrashing rates were scored on day one of adulthood for WT **(C)** or *hlh-1(ts)* **(D)** animals grown at 15°C for the duration of the experiment (Ctrl-75 h) or shifted to 25°C at the indicated times (I-15h, II-36h, III-46 h or IV-60 h). Data are means ± 1 standard error of the mean (1SE). Data were analyzed using one-way ANOVA followed by a Dunnett’s post-hoc test (N = 3, *n* = 30). (*) denotes *p* < 0.05, (**) denotes *p* < 0.01, (***) denotes *p* < 0.001, and (ns) denotes *p* > 0.05, compared with Ctrl animals maintain at 15°C. **(E)** Representative images of age-synchronized *hlh-1(ts)* animals that express MYO-3::GFP. Animals were grown at 15°C for the duration of the experiment (Ctrl) or shifted to 25°C at L1-I or L2-II. Animals were collected and fixed at the YA stage, and myofilaments were imaged. The scale bar is 25 μm. Panels A–B were created using BioRender.com.

Nematodes were grown on NGM plates seeded with the *Escherichia coli* OP50-1 strain and maintained at 15°C. Age-synchronized embryos were obtained by placing for 3–5 h ∼25 adults on fresh plates at 15°C, as in (Dror et al., 2020). Nematodes were either maintained at 15°C for the duration of an experiment or shifted to 25°C at specific time points during development (Figures 1A,B). Unless otherwise stated, animals’ motility, muscle organization, or gene expression were examined on the first day of adulthood before the onset of egg-laying (young adults, YA). A list of strains used in this work is included in Supplementary Table S1. All strains were outcrossed to our N2 stock at least four times. Cross-strains were generated using standard *C. elegans* procedures. Animals’ genotype was confirmed by single worm PCR using Phire Animal Tissue Direct PCR Kit (F-170L, Thermo Scientific) with primers that amplified the area of the mutation as in (Meshnik et al., 2022). Primer sequences used in this study are listed in Supplementary Table S2.

### 2.2 Temperature Shift Experiments

Wild type (WT), *hlh-1(cc561), smg-6(*ok1794), or *hlh-1(cc561);smg-6(ok1794)* embryos laid at 15°C were shifted to 25°C at the first (L1, I), second (L2, II), third (L3, III), or fourth (L4, IV) larval stage and maintained at 25°C until they reached the YA stage. As a control (Ctrl), embryos laid at 15°C were allowed to develop at 15°C until the YA stage (Figure 1A). YA motility, muscle organization, or mRNA levels were then examined. Cultivation times at 15°C and 25°C for each experimental condition are noted (Figure 1B). We treated all examined strains in parallel for each experimental condition when comparing different strains.

### 2.3 Motility Assay

To determine motility rates, we counted the number of changes in bending direction at mid-body (thrashes) per minute of age-synchronized animals (N = 3, *n* = 30), as in (Dror et al., 2020).

### 2.4 Feeding RNA Interference

Synchronized embryos (N = 3, *n* > 30) were placed on plates seeded with *E. coli* strain HT115 (DE3) transformed with the indicated RNA interference (RNAi) vectors, *smg-2* or *smg-7*, (obtained from the Vidal RNAi library) or empty vector (EV) control (pL4440), as previously described (Dror et al., 2020). The efficiency of the RNAi treatment was determined using qPCR to examine the mRNA levels of *smg-2* or *smg-7*. The efficiency of mRNA knockdown ranged between 50–90%.

### 2.5 Confocal Imaging

Adult animals expressing MYO-3 tagged with GFP (strains DM8005 or RW1596) were fixed with 4% paraformaldehyde, as previously described (Karady et al., 2013). Alternatively, adult animals were fixed with 4% paraformaldehyde, permeabilized with ice-cold acetone, and stained with Rhodamine-Phalloidin (1:10; 00027, Biotium). Treated samples were imaged using a Leica DM5500 confocal microscope through a 63x 1.0 numerical aperture objective with a 488 nm or 532 lines for excitation, respectively.

### 2.6 RNA Levels

RNA was extracted from age-synchronized animals using GENEzol TriRNA Pure Kit (GZXD200, Geneaid), and was then treated with DNA Free^TM^ DNA removal kit (AM 1906, Invitrogen). For cDNA synthesis, RNA was reverse-transcribed using the iScript^TM^ cDNA Synthesis Kit (1708891, Bio-Rad). mRNA levels were measured using quantitative PCR, performed on a C1000 Thermal Cycler (Bio-Rad) with KAPA SYBRFAST qPCR Master Mix (KK4602, KAPA Biosystems), as in (Meshnik et al., 2022). Relative transcript levels were determined by averaging the C_T_ of triplicate values for the genes examined and normalizing those to C_T_ values obtained for 18S rRNA of the same sample using the 2^−ΔΔCT^_T_ method (Livak and Schmittgen, 2001). List of primes used in this work are provided in Supplementary Table S2.

### 2.7 Statistical Analysis

**FIGURE 2:**
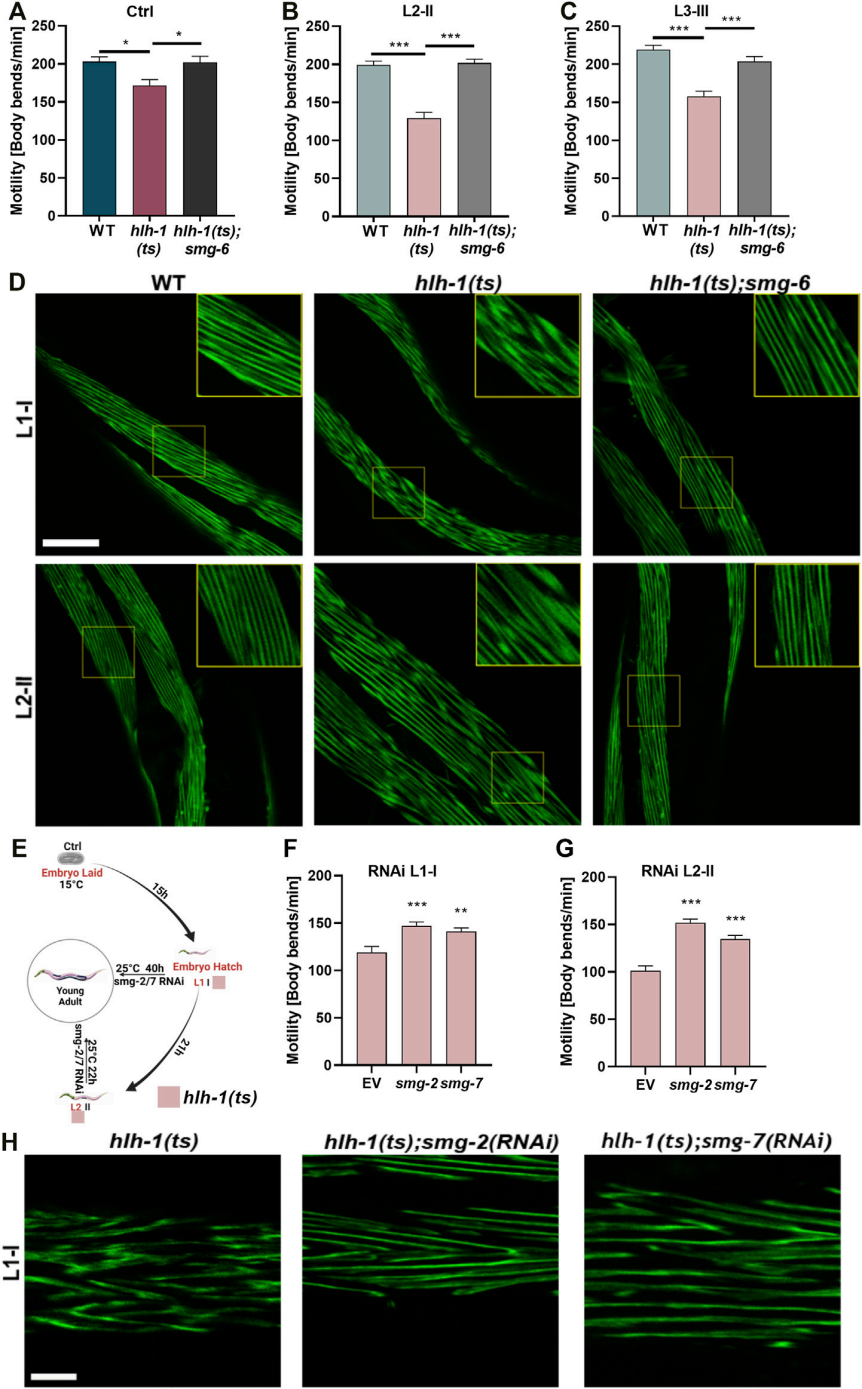
Blocking the RNA-mediated decay pathway rescues animals’ motility. **(A–C)** Motility rates of wild type (WT), *hlh-1(ts),* or *hlh-1(ts);smg-6*. Thrashing rates of age-synchronized WT, *hlh-1(ts),* or *hlh-1(ts);smg-6* grown at 15°C for the experiment duration **(A)** or shifted to 25°C at L2 [II, **(B)**] or L3 [III, **(C)**] larval stages. Data are means ± 1 standard error of the mean (1SE). Data were analyzed using one-way ANOVA followed by a Tukey’s post hoc test (N = 3, *n* = 20). (*) denotes *p* < 0.05, and (***) denotes *p* < 0.001 compared with WT. **(D)** Representative images of temperature-shifted animals expressing MYO-3:GFP. WT, *hlh-1(ts)*, or *hlh-1(ts);smg-6* animals were shifted to 25°C at L1-I or L2-II. YA were collected and fixed, and myofilaments were imaged. The scale bar is 25 μm. Inserts are a 2-fold magnification of the boxed area. **(E)** Schematic representation of the experimental setup. *hlh-1(ts)* embryos were laid on seeded plates at 15°C. Age-synchronized *hlh-1(ts)* animals were moved to RNAi plates seeded with *smg-2*, *smg-7*, or empty vector (EV) control, and plates were shifted to 25°C at L1 (I) or L2 (II) larval stages. YAs were then analyzed for motility and myofilament organization. **(F–G)** Motility rates of *hlh-1(ts) smg(RNAi)* treated animals. Thrashing rates of age-synchronized *hlh-1(ts*) animals that were moved to RNAi plates seeded with *smg-2*, *smg-7*, or EV control and shifted to 25°C at L1 **(F)** or L2 **(G)** larval stages. Data are means ± 1 standard error of the mean (1SE). Data were analyzed using one-way ANOVA followed by a Dunnett’s post hoc test (N = 3, *n* = 60). (**) denotes *p* < 0.01 and (***) denotes *p* < 0.001 compared with EV control. **(H)** Representative images of *hlh-1(ts) smg(RNAi)* treated myofilament. Age-synchronized *hlh-1(ts)* animals expressing MYO-3:GFP were treated with *smg-2*, *smg-7*, or EV control RNAi at 25°C as in G, and myofilaments were imaged. The scale bar is 7.5 μm. Panel E was created using BioRender.com.

**FIGURE 3:**
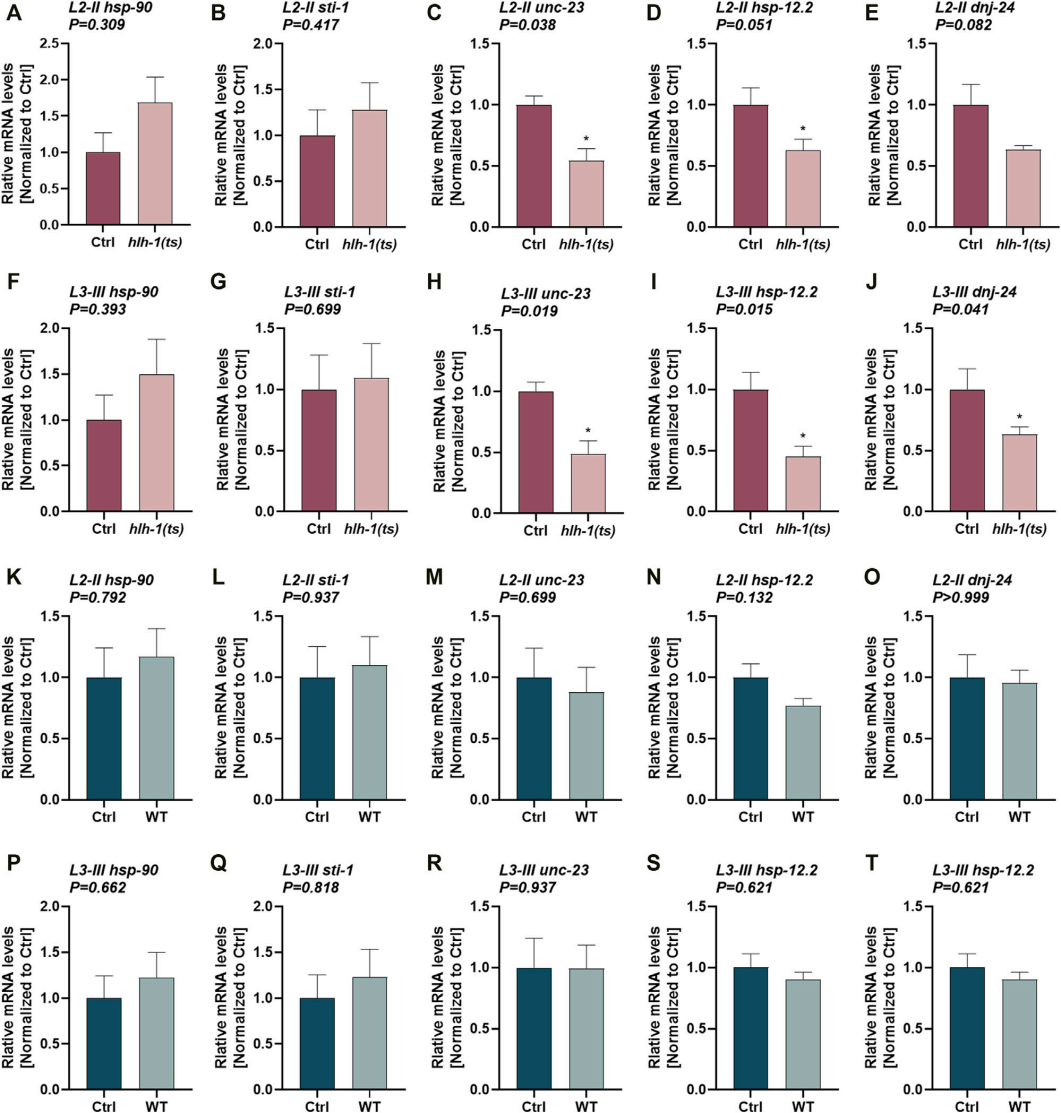
The expression of HLH-1-dependent chaperones during larval development. **(A–T)** Expression levels of chaperones in *hlh-1(ts)* animals. Relative mRNA levels of HLH-1-dependent chaperones, *hsp-90*, *sti-1*, *unc-23*, *hsp-12.2*, or *dnj-24*, in age synchronized *hlh-1(ts)* **(A–J)** or WT **(K–T)** animals, shifted to 25°C at L2-II [**(A-E)** and **(K-O)**] or L3-III [**(F-J)** and **(P-T**)] larval stages. Expression levels were compared with the same strain animals maintained at 15°C (Ctrl). Data are means ± 1 standard error of the mean (1SE). Data were analyzed using the Wilcoxon Mann-Whitney rank sum test compared with the same strain Ctrl treated animals (N ≥ 4).

**FIGURE 4:**
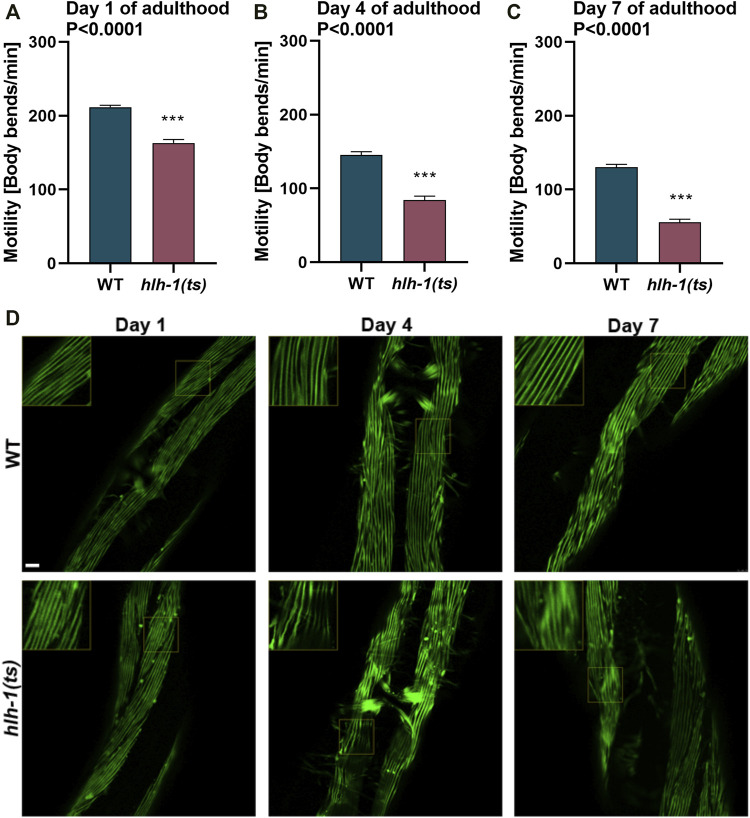
Mild disruption of *hlh-1* expression during development severely impact muscle maintenance later in adulthood. **(A–C)** Motility rates of WT or *hlh-1(ts)* adults. Thrashing rates of age-synchronized WT or *hlh-1(ts)* animals maintained at 15°C were examined on days 1 **(A)**, 4 **(B)**, or 7 **(C)** of adulthood. Data are means ± 1 standard error of the mean (1SE). Data were analyzed using the Wilcoxon Mann-Whitney rank sum test compared with WT (N = 3, *n* ≥ 30). **(D)** Representative images of WT or *hlh-1(ts)* animals that express MYO-3::GFP. Age-synchronized WT or *hlh-1(ts)* animals maintained at 15°C were collected and fixed on days 1, 4, or 7 of adulthood, and myofilaments were imaged. Inserts are a 2-fold magnification of the boxed area. The scale bar is 10 μm.

To test the null hypothesis that *hlh-1* or chaperone mRNA levels are reduced in *hlh-1(ts)* animals compared to WT (Supplementary Figures S1A, S4C), we used a one-way analysis of variance (ANOVA) followed by Bonferroni’s post hoc test. We used one-way ANOVA followed by Dunnett’s post hoc test to compare motility rates or mRNA levels between Ctrl and temperature shifted animals (Figures 1C,D and Supplementary Figure S3U–W, respectively). We used the same test to compare motility rates between *smg-2*, or *smg-7* RNAi treated animals and EV control at different knockdown stages (Figures 2F,G). We used one-way ANOVA followed by Tukey’s post hoc test to compare motility rates between WT, *hlh-1(cc561),* or *hlh-1(cc561);smg-6(ok1794)* mutant animals treated as Ctrl, L2-II or L3-III (Figures 2A–C). We used the Wilcoxon Mann-Whitney rank sum test to ask whether muscle chaperone expression levels are reduced in *hlh-1*(ts) compared to WT animals (Figure 3 and Supplementary Figure S3A–T) or to ask whether *hlh-1* levels are elevated in *hlh-1(ts);smg-6* mutant animals treated as Ctrl, L2-II or L3-III compared to *hlh-1(ts)* or WT (Supplementary Figure S2). The same test was used to ask whether the motility of *hlh-1(ts)* animals was reduced compared to WT animals maintained at 15°C at different larval and adult stages (Figures 4A–C and Supplementary Figure S4A–B). Bonferroni correction was applied to adjust *p* values when gene expression was compared under two conditions. Data are presented as bar graphs showing means ± 1 standard error of the mean (1SE). The numbers of biological repeats (N) and individuals (n) in each condition tested are noted in the figure legends.

## 3 Results

### 3.1 HLH-1 is Required for Muscle Maintenance During Larval Development

*hlh-1* is expressed during larval development, with mRNA and protein expression detected in all larval stages (L1-L4) (Harfe et al., 1998; Toudji-Zouaz et al., 2021). During early larval development (L1-L2), HLH-1 plays a role in postembryonic muscle differentiation of the 14 body-wall muscle cells derived from the M-lineage (Harfe et al., 1998; Krause and Liu, 2012). In agreement, we detected *hlh-1* mRNA expression in larvae. This expression declined with age (Supplementary Figure S1) (Gerstein et al., 2014).

To ask whether HLH-1 affects muscle postembryonic maintenance, we utilized a nonsense mutant allele, *hlh-1(cc561ts)* (hereafter designated as *hlh-1(ts)*)*,* that gives rise to a truncated protein. The phenotype is attributed to insufficient HLH-1 levels, as overexpression of the truncated mRNA or disruption of the nonsense-mediated decay (NMD) pathway rescues *hlh-1(ts)* phenotypes. The *hlh-1(ts)* mutation behaves like a conditional knockdown, likely because the nonsense-mediated decay pathway activity is modulated by temperature (Harfe et al., 1998; Cali et al., 1999). Whereas the animals appear wild-type at low cultivation temperatures, and there is minimal impact on *hlh-1* levels (Supplementary Figure S1), motility, and body wall muscles organization (Gieseler et al., 2000; Gieseler et al., 2002). The mutation is fully evident at high cultivation temperatures, showing defective embryonic and postembryonic muscle differentiation and L1-arrest phenotype (Harfe et al., 1998). We thus used *hlh-1(ts)* temperature-sensitive behavior to knock down *hlh-1* levels at different stages during larval development and examine its impact on muscle maintenance.

Age synchronized wild-type (WT) or *hlh-1(ts)* embryos were allowed to hatch and develop under permissive conditions (15°C). Animals were shifted to the restrictive conditions (25°C) at first (L1, I), second (L2, II), third (L3, III), or fourth (L4, IV) larval stages (Figure 1A) and then maintained under restrictive conditions for the duration of the experiment (Figure 1B). The motility of temperature-shifted young adult animals (YA), measured as thrashing rates, was compared to that of YA animals maintained at the permissive conditions for the duration of the experiment (Ctrl, Figures 1A,B). Whereas the motility of WT larvae shifted to the restrictive conditions was unaffected, motility of *hlh-1(ts)* animals shifted at L1, L2, or L3, but not L4, was significantly reduced (1.8-, 1.4-, and 1.2-fold, respectively, ANOVA followed by a Dunnett’s post hoc test, Figures 1C,D). Thus, disrupting *hlh-1* expression during larval development affects muscle function, supporting a postembryonic role for HLH-1 in muscle maintenance.

The postembryonic differentiation of M-lineage derived body-wall muscles can occur in the absence of HLH-1, and it is completed before the L3 stage (Harfe et al., 1998; Krause and Liu, 2012). To determine if the impact of *hlh-1* knockdown on motility was specific to the M-derived body-wall muscle cells or general, we examined whether and which body-wall muscle cells display disrupted myofilament organization. For that, we monitored myosin heavy chain A (MYO-3) organization using an MYO-3::GFP tagged protein. L1-or L2-shifted *hlh-1(ts)* animals (L1-I and L2-II, respectively) exhibited disrupted myofilament structure and MYO-3::GFP mislocalization in most muscle cells, while animals maintained at permissive conditions were unaffected (Figure 1E). Thus, *hlh-1* is required to maintain embryonic and postembryonic differentiated body-wall muscles.

### 3.2 Restoring *hlh-1* mRNA During Development Rescues Muscle Proteostasis

We next asked whether inhibiting the NMD pathway can restore muscle maintenance by disrupting the expression of the *smg* mRNA surveillance genes, using animals harboring a mutation in *smg-6(ok1794)* (hereafter designated as *smg-6*). Double mutant *hlh-1(ts); smg-6* animals that were maintained under permissive conditions (Ctrl) showed no or a mild increase in *hlh-1* mRNA levels compared to *hlh-1(ts)* or WT animals (Wilcoxon Mann-Whitney rank sum test, *p* = 0.2, and *p* = 0.028, respectively; Supplementary Figure S2A–B). In agreement, the mild motility reduction (1.15-fold) of Ctrl treated *hlh-1(ts)* animals compared to WT animals was abolished in the double mutant *hlh-1(ts); smg-6* animals (ANOVA followed by a Tukey’s post hoc test, *p* ≤0.05; Figure 2A). This behavior was more apparent in temperature-shifted animals than in Ctrl-treated animals. *hlh-1* mRNA levels in animals shifted at L2-II or L3-III were significantly higher for *hlh-1(ts);smg-6* than for *hlh-1(ts)* mutant or WT animals, restoring and even raising *hlh-1* mRNA levels (Wilcoxon Mann-Whitney rank sum test, *p* = 0.028; Supplementary Figure S2C–F). Likewise, the reduced motility of L2-or L3-shifted *hlh-1(ts)* animals compared to WT (129 ± 8 vs. 199 ± 5 bends per minute and 158 ± 7 vs. 219 ± 5 bends per minute, respectively) was rescued in *hlh-1(ts);smg-6* shifted animals (202 ± 5 and 204 ± 6 bends per minute, respectively, ANOVA followed by a Tukey’s post hoc test, *p* ≤0.001; Figures 2B,C). Furthermore, the severe MYO-3::GFP mislocalization observed in *hlh-1(ts)* animals shifted at L1-I or L2-II was abrogated in *hlh-1(ts);smg-6* animals (Figure 2D). Thus, the observed developmental muscle maintenance phenotypes are dependent on HLH-1.

One possible interpretation of our results is that the mild reduction in *hlh-1* mRNA levels during embryogenesis alone gives rise to the muscle defects observed during development under permissive conditions. We thus asked whether inhibiting the NMD pathway past embryogenesis can still rescue muscle defects in temperature-shifted larvae to address this possibility. We knocked down the expression of *smg* genes during larval development by introducing RNAi against *smg-2* or *smg-7* only once we shifted the animals to the restrictive conditions (Figure 2E). The motility of *hlh-1(ts)* temperature-shifted L1-I or L2-II animals treated with *smg-2(RNAi)* or *smg-7(RNAi)* was significantly improved compared to animals treated with empty vector (EV) control (∼1.4- and ∼1.2-fold, respectively, ANOVA followed by a Dunnett’s post hoc test, *p* ≤0.01; Figures 2F,G). Likewise, MYO-3:GFP mislocalization observed in EV control-treated *hlh-1(ts)* L1-I shifted animals was strongly reduced in *smg-2(RNAi)* or *smg-7(RNAi)* treated L1-I shifted animals (Figure 2H). Taken together, HLH-1 is required for muscle maintenance during larval development.

### 3.3 HLH-1 Knockdown During Larval Development Modulates Muscle Chaperone Expression

MyoD/HLH-1 can regulate chaperone expression during muscle differentiation (Sugiyama et al., 2000; Bar-Lavan et al., 2016b; Echeverria et al., 2016; Tiago et al., 2021). To ask whether HLH-1 also regulates chaperones’ expression during larval development, we examined the expression of chaperones that are *hlh-1*-dependent or *hlh-1*-independent. While *hlh-1*-dependent chaperones were shown to have putative HLH-1 occupancy sites at their promoter and respond to modulation of *hlh-1* expression levels in embryos, *hlh-1*-independent chaperones lack such features. We first compared mRNA levels of chaperones in Ctrl and L2-II and L3-III temperature-shifted *hlh-1(ts)* (Figures 3A–J) or WT (Figure 3K-T) animals using qPCR. *unc-23*, *hsp-12.2*, and *dnj-24* showed reduced expression in L2-II (Figures 3C–E) and L3-III (Figures 3H–J) temperature-shifted *hlh-1(ts)* animals compared to animals maintained under permissive conditions, but not in WT animals (Wilcoxon Mann-Whitney rank sum test; Supplementary Figure S3M–O, R–T). *unc-23*, *hsp-12.2*, and *dnj-24* reduced expression was rescued in temperature-shifted *hlh-1(ts); smg-6* animals (ANOVA followed by a Dunnett’s post hoc test; Supplementary Figure S3U–W), when *hlh-1* levels are elevated (Supplementary Figure S2C–F). The expression levels of the other two *hlh-1*-dependent chaperones, *hsp-90* and *sti-1*, were not significantly different (Wilcoxon Mann-Whitney rank sum test). *hsp-90* and *sti-1* are highly and ubiquitously expressed chaperones, and changes in their expression in one tissue might not be apparent. While *unc-23*, *hsp-12.2*, and *dnj-24* are also expressed in other tissues, their expression is enriched in muscle cells (Roy et al., 2002; Fox et al., 2007; Kuntz et al., 2012). Indeed, their expression pattern clusters with muscle-specific genes during embryogenesis, showing a myogenesis-induced expression pattern (while *hsp-90* and *sti-1* do not) (Bar-Lavan et al., 2016b). In contrast, when we examined the expression levels of five *hlh-1*-independent chaperones (Supplementary Figure S3), only *hsp-17* showed reduced expression in temperature-shifted *hlh-1(ts)* animals (Supplementary Figure S3A, F). However, it was similarly affected in temperature-shifted WT animals (Wilcoxon Mann-Whitney rank sum test; Supplementary Figure S3K, P). Thus, *hlh-1*-dependent chaperone expression can be modulated by *hlh-1* knockdown during larval development, suggesting that HLH-1 could contribute to muscle proteostasis.

### 3.4 Disruption in *hlh-1* Expression During Embryonic or Larval Development Results in Early Onset of Muscle Deterioration

RNAi knockdown of *hlh-1* in adulthood shows no impact on muscle function in adulthood (Mergoud Dit Lamarche et al., 2018). In agreement, the motility of L4 temperature-shifted *hlh-1(ts)* animals was similar to animals grown under permissive conditions (Figure 1D). These data suggest that HLH-1 has no role in muscle maintenance during adulthood. However, *hlh-1* function during embryonic and larval development could impact muscle proteome and proteostasis and thus modulate muscle maintenance later in life. To address this possibility, we ask whether the mild reduction in *hlh-1* mRNA levels (Supplementary Figure S1) and its’ associated phenotypes (Figure 1E, 2A, and Supplementary Figure S4A–C) observed in *hlh-1(ts)* animals maintained at permissive conditions has implications on muscle maintenance in adulthood. The motility of *hlh-1(ts)* animals was mildly reduced compared to WT during larval development (∼1.15-fold, Wilcoxon Mann-Whitney rank sum test; Supplementary Figure S4A–B). However, with age, motility decline became more pronounced (Figures 4A–C). By day four of adulthood, *hlh-1(ts)* animals’ thrashing rate was reduced by 1.7-fold compared to WT (Wilcoxon Mann-Whitney rank sum test, *p* < 0.001; Figure 4B). Motility declined further by day 7 of adulthood (2.3-fold compared to WT, Wilcoxon Mann-Whitney rank sum test, *p* < 0.0001; Figure 4C). Of note, while WT motility declined by 1.6-fold with age, *hlh-1(ts)* motility declined by 3-fold. Likewise, myofilament organization was disrupted with age. MYO-3 and actin filaments disruption was apparent already at day 4 of adulthood. By day 7 of adulthood, MYO-3 and actin organization were disrupted in most body wall muscle cells (Figure 4D and Supplementary Figure S4D). In contrast, MYO-3 organization in WT animals remained intact, as previously observed (Ben-Zvi et al., 2009). Thus, the mild decline in *hlh-1* expression levels associated with mild or no changes in muscle maintenance during larval development (Figures 1E,F, 2A, and Supplementary Figure S4A–C) is sufficient to disrupt the muscle folding environment and thus muscle maintenance in adulthood.

## 4 Discussion

Chaperone expression is differentially regulated across cell types during cellular differentiation to establish tissue-specific chaperone networks (Nisaa and Ben-Zvi, 2021; Shemesh et al., 2021). Differentiation transcription factors play a role in the rewiring of the chaperone network in parallel to setting the cellular proteome (Luo et al., 1991; Bar-Lavan et al., 2016b; Piri et al., 2016; Zha et al., 2019; Nisaa and Ben-Zvi, 2021). Specifically for muscle cells, MyoD/HLH-1 was shown to directly regulate chaperone expression (Sugiyama et al., 2000; Bar-Lavan et al., 2016b; Echeverria et al., 2016; Tiago et al., 2021). Tissue-specific chaperone networks are also maintained in adult tissues and deteriorate with age (Brehme et al., 2014; Sala et al., 2017; Shemesh et al., 2021). Here, we asked whether a differentiation transcription factor can be involved in muscle maintenance later in life. To address this question, we set out to examine the effects of conditional knockdown of *C. elegans* MyoD, HLH-1, at different stages of larval development.

We first established that *hlh-1* is expressed during larval development and is required for motility and muscle organization, although the effect was most pronounced in early larval stages (Figures 1, 2). Furthermore, we showed that disrupting *hlh-1* expression during L2-L3 larval stages resulted in reduced expression of some HLH-1-dependent chaperones but not HLH-1-independent chaperones (Figure 3). Finally, we showed that even a mild reduction in *hlh-1* expression during embryogenesis and larval development with no or mild impact on motility and muscle organization in YA showed faster deterioration of muscles during aging (Figure 4). Taken together, we propose a role for MyoD/HLH-1 in the maintenance of muscle proteostasis.

### 4.1 HLH-1-dependent Chaperones Shape the Muscle Chaperone Folding Capacity

Muscle chaperones, most of which are *hlh-1-*dependent, are required for muscle proteostasis (Bar-Lavan et al., 2016b; Nisaa and Ben-Zvi, 2021; Shemesh et al., 2021). Disrupting the expression of a single muscle chaperone by knockdown or overexpression often affects muscle proteome folding and function and, for some chaperones, can even disrupt muscle differentiation (Landsverk et al., 2007; Frumkin et al., 2014; Papsdorf et al., 2014; Bar-Lavan et al., 2016b; Echeverria et al., 2016; Nisaa and Ben-Zvi, 2021; Tiago et al., 2021). For example, DNJ-24, UNC-23, HSP-90, and STI-1 are localized to the sarcomere, and when any are disrupted, myosin is disorganized (Meissner et al., 2011; Frumkin et al., 2014; Papsdorf et al., 2014; Rahmani et al., 2015; Bar-Lavan et al., 2016b). Indeed, when the functional association of chaperones that are upregulated in human skeletal muscle (and their *C. elegans* homologs) was examined, most were required for muscle function, and more than half were causal or associated with muscle diseases (Shemesh et al., 2021). For example, mutations in DNAJB6 (homolog of *dnj-24*) lead to Limb-girdle muscular dystrophy 1E, and mutations in HSPB8 (homolog of *hsp-12.2*) or BAG2 (homolog of *unc-23*) were associated with myopathies (Sarparanta et al., 2012; Al-Tahan et al., 2019; Diofano et al., 2020). Moreover, the knockdown of muscle chaperone expression activated a muscle-specific stress response (Guisbert et al., 2013). The tight regulation of muscle chaperones is most apparent when overexpression of chaperones, including HSP-90, HSP-1, and UNC-45, disrupt, rather than improve, muscle proteostasis (Landsverk et al., 2007; Papsdorf et al., 2014; Bar-Lavan et al., 2016b; Echeverria et al., 2016; Nisaa and Ben-Zvi, 2021). Thus, MyoD/HLH-1 modulates the regulation of muscle chaperones. In turn, muscle chaperones directly contribute to muscle health, supporting a role for MyoD/HLH-1 in muscle-specific regulation of chaperone expression to address the specific folding needs of the muscle proteome.

### 4.2 The Function of HLH-1 During Embryonic and Larval Development Shapes Muscle Proteostasis in Adulthood

*hlh-1* knockdown in adulthood has no impact on muscle maintenance later in life, suggesting that HLH-1 mainly functions in embryos and developing larvae (Mergoud Dit Lamarche et al., 2018). This developmental role is supported by the observed decline in *hlh-1* requirement during larval development (Figure 1D) and the low *hlh-1* levels detected in young adults (Supplementary Figure S1). While it is likely that HLH-1 has no active role in muscle maintenance during adulthood, even mild disruption in *hlh-1* expression during larval development strongly impacted muscle maintenance in adulthood (Figure 4 and Supplementary Figure S4). The impact of *hlh-1* disruption suggests that the basal state of muscle proteostasis early in adulthood can substantially affect muscle maintenance in aging. In agreement, aggregation prone poly-glutamine extended repeat model (Q35) in *hlh-1(ts)* background show increase aggregation rate mainly in adulthood (Bar-Lavan et al., 2016b). A model for Duchenne muscular dystrophy (DMD), consisting of animals carrying a null mutation in dystrophin in *hlh-1(ts)* background, showed similar behavior. Animals displayed a progressive loss of motility and muscle degeneration. Disruption-associated damage that started in development became functionally apparent during *C. elegans* adulthood (Gieseler et al., 2000; Gieseler et al., 2002; Brouilly et al., 2015) as well as in mice (Megeney et al., 1999). Several studies demonstrated a role for proteostasis maintenance in this *C. elegans* DMD model (Nyamsuren et al., 2007; Brouilly et al., 2015)*.* Specifically, deletion of the co-chaperone, STUB1 (*chn-1* in *C. elegans*), or inhibition of the proteasome partially rescued muscle function and structure in adulthood, delaying muscle wasting (Nyamsuren et al., 2007). Muscle-specific gene expression analyses comparing WT and dystrophin mutants after phenotypes were observed (L3-adult) identified significant changes in the expression of genes involved in myogenesis, supporting a role for HLH-1 in muscle maintenance (Hrach et al., 2020). We, therefore, propose that the basal proteostasis network in *hlh-1(ts)* mutant animals is limiting, which contributes to age-dependent muscle deterioration, accelerating the rate of muscle wasting.

### 4.3 Various Transcription Factors can Shape the Cell-specific Proteostasis Networks

Three myogenic transcription factors are involved in muscle differentiation during embryogenesis, HLH-1, UNC-120, and HND-1 (Fukushige and Krause, 2005; Fukushige et al., 2006; Lei et al., 2010; Kuntz et al., 2012). Like HLH-1, UNC-120 is expressed past embryogenesis and protects muscle cells later in life (Mergoud Dit Lamarche et al., 2018). Specifically, UNC-120 knockdown results in reduced expression of muscle genes, disrupted mitochondria morphology and connectivity, and accumulation of autophagic vesicles, suggesting that UNC-120 impacts muscle health. In agreement, UNC-120 overexpression in body wall muscle cells improves muscle aging. Moreover, UNC-120 can affect the expression of some chaperones (Kuntz et al., 2012), suggesting that, like HLH-1, it can shape muscle chaperone network in adulthood.

Other transcription factors, such as the heat shock transcription factors (HSFs), regulate chaperones’ expression during development (Li et al., 2016; Li et al., 2017) or modulate the muscle heat shock response (Guisbert et al., 2013). Furthermore, trans-cellular signaling modulates chaperone expression in muscle in response to changes in chaperone expression in neurons or intestine cells (O'Brien et al., 2018; Miles et al., 2022). Therefore, the chaperone system can be shaped and reshaped during development. Different transcription factors can contribute to the composition, expression levels, and thus folding capacity of muscle cells in development and aging. How these signals regulate and coordinate an intricate chaperone network that responds to tissue and organismal folding demands remains to be determined.

## Data Availability

The original contributions presented in the study are included in the article/Supplementary Material, further inquiries can be directed to the corresponding author.
