# Supplementary material for: HLH-1 Modulates Muscle Proteostasis During Caenorhabditis elegans Larval Development
Source: Front Cell Dev Biol. 2022 Jun 6;10:920569. doi: 10.3389/fcell.2022.920569 (PMC9207508; doi:10.3389/fcell.2022.920569)
Supplement: Supplementary file 1 [file DataSheet1.pdf]

Supplementary Material

HLH-1 modulates muscle proteostasis during *Caenorhabditis elegans* larval development

Khairun Nisaa, Anat Ben-Zvi

1 Supplementary Figures

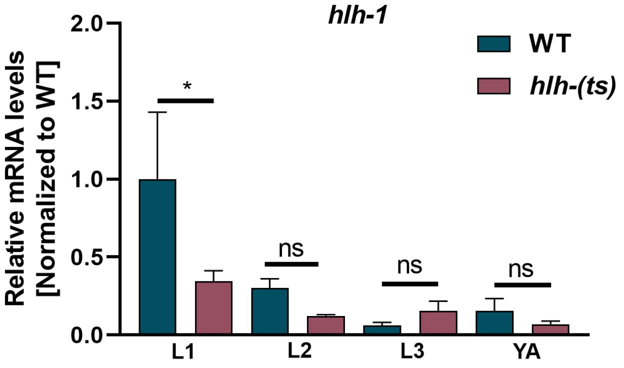

Supplementary Figure 1. *hlh-1* is expressed during *C. elegans* larval development. *hlh-1* expression during larval development. mRNA levels of *hlh-1* from age-synchronized wild type (WT) or *hlh-1(ts)* animals grown under permissive conditions (15°C) at the indicated larval stages (N=7). Data are means ± 1 standard error of the mean (1SE). Data were analyzed using one-way ANOVA followed by Bonferroni's post hoc test. (*) denotes *P*< 0.05, and (ns) denotes P> 0.05 compared with same stage WT animals.

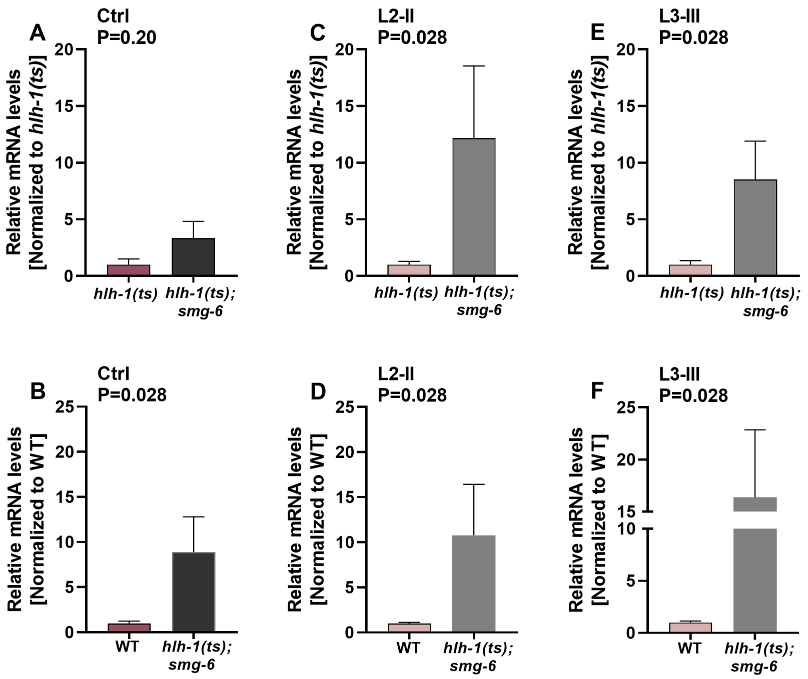

Supplementary Figure 2. Downregulation of the NMD pathway results in the elevation of *hlh-1* mRNA levels. (A-F) Rescue of *hlh-1* levels. Relative mRNA levels of *hlh-1* in age-synchronized *hlh-1(ts)* (A, C and E) or WT (B, D and F) compared to *hlh-1(ts);smg-6* mutant animals grown at 15°C (Ctrl, *P*=0.2 and *P*=0.028, respectively; A-B), or shifted to 25°C at L2 (II, *P*=0.028; C-D) or L3 (III, *P*=0.028; E-F) larval stages. Data are means ± 1 standard error of the mean (1SE). Data were analyzed using the Wilcoxon Mann-Whitney rank sum test (N=4).

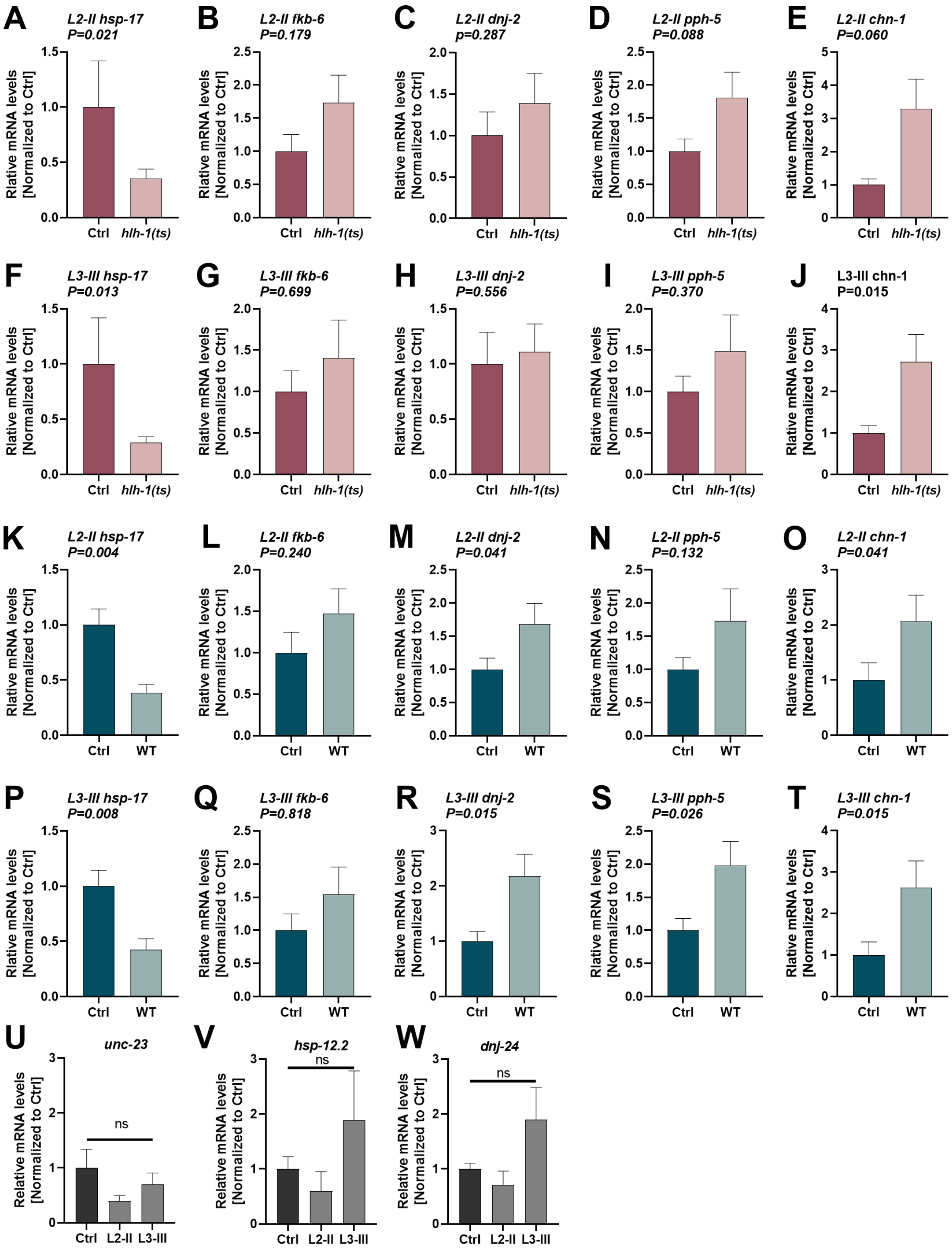

Supplementary Figure 3. Expression of chaperones during larval development. (A-T) Expression of HLH-1-independent chaperones. Relative mRNA levels of HLH-1-independent chaperones, *hsp-17*, *fkb-6*, *dnj-2*, *pph*-5, or *chn-1*, in age synchronized *hlh-1(ts)* (A-J) or WT (K-T) animals, shifted to 25°C at L2-II (A-E and K-O) or L3-III (F-J and P-T) larval stages. Levels were compared with same strain animals maintained at 15°C (Ctrl). Data are means ± 1 standard error of the mean (1SE). Data were analyzed using the Wilcoxon Mann-Whitney rank sum test compared with Ctrl (N≥4). (U-W) NMD-dependent rescue of chaperone expression levels. Relative mRNA levels of HLH-1-dependent chaperones, *unc-23* (U), *hsp-12.2* (V), or *dnj-24* (W), in age synchronized *hlh-1(ts);smg-6* animals, shifted to 25°C at L2-II or L3-III larval stages. Expression levels were compared with same strain animals maintained at 15°C (Ctrl). Data are means ± 1 standard error of the mean (1SE). Data were analyzed using one-way ANOVA followed by a Dunnett's post hoc test compared with Ctrl (N=5).

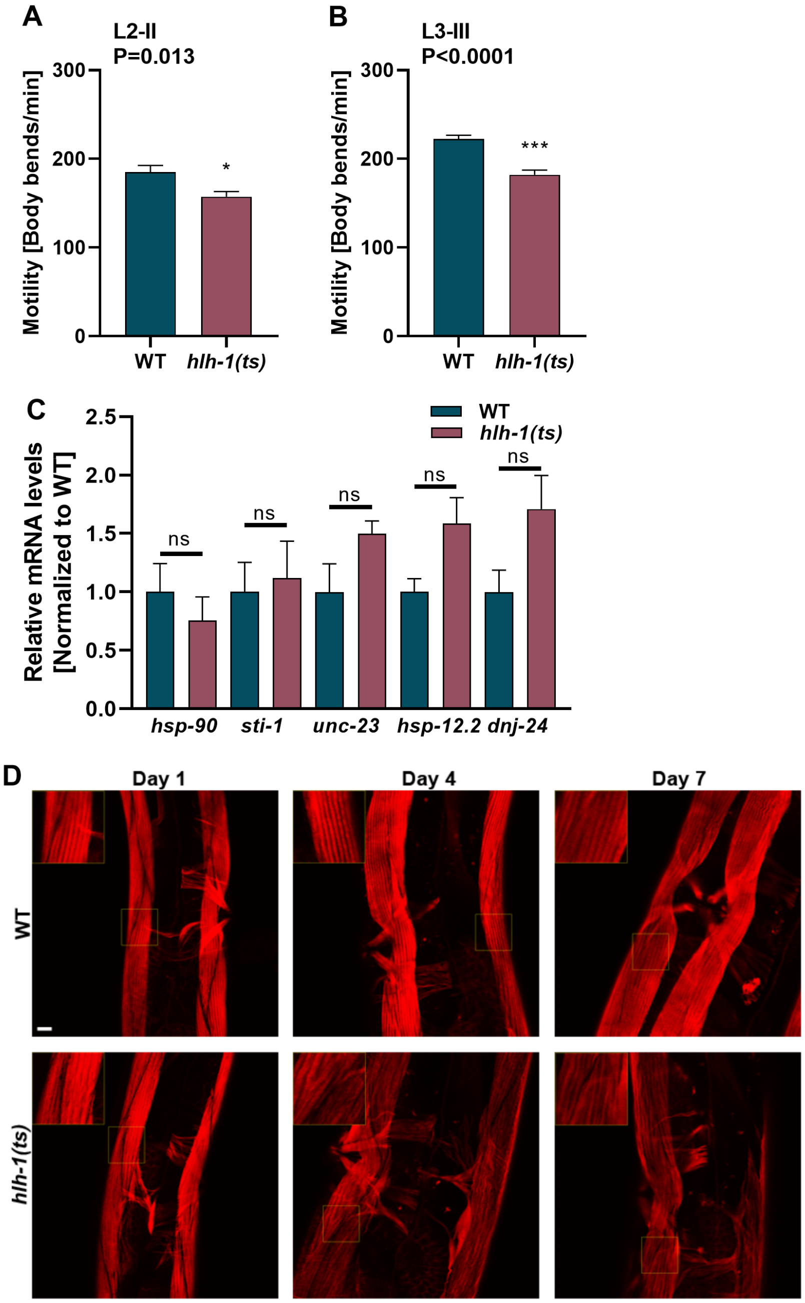

Supplementary Figure 4. *hlh-1(ts)* impact *C. elegans* larval development. (A-B) Motility rates of WT or *hlh-1(ts)* larvae at 15°C. Thrashing rates of age-synchronized wildtype or *hlh-1(ts)* animals grown at 15°C were scored at L2 (A) or L3 (B). Data were analyzed using the Wilcoxon Mann-Whitney rank sum test (N=3, n=30). (C) Expression levels of chaperones in WT and *hlh-1(ts)* animals. Relative mRNA levels of HLH-1-dependent chaperones, *hsp-90*, *sti-1*, *unc-23*, *hsp-12.2*, or *dnj-24*, in age-synchronized WT or *hlh-1(ts)* animals grown at 15°C. Data are means ± 1 standard error of the mean (1SE). Data were analyzed using one-way ANOVA followed by Bonferroni's post hoc test (N≥4). (ns) denotes P> 0.05 compared with WT animals grown at 15°C. (D) Representative images of WT or *hlh-1(ts)* actin filaments. Age-synchronized WT or *hlh-1(ts)* animals maintained at 15°C were collected and fixed on days 1, 4, or 7 of adulthood, and actin filaments were imaged. The scale bar is 10 μm.

2 Supplementary Tables

Strain	Abbreviation	Genotype
**N2**	wild type (WT)	---
**PD4605**	*hlh-1(ts)*	*hlh-1(cc561)*
**VC1305**	*smg-6*	*smg-6(ok1974)*
**RW1596**	MYO-3::GFP	*stEx30[myo-3p::GFP::myo-3 + rol-6(su1006)]*
**DM8005**	MYO-3::GFP	*raIs5[myo-3p::GFP::myo-3 + rol-6(su1006)]*
**ABZ240**	*hlh-1(ts);smg-6*	*hlh-1(cc561);smg-6(ok1794)*
**ABZ241**	*hlh-1(ts);*MYO-3::GFP	*hlh-1(cc561); raIs5[myo-3p::GFP::myo-3 + rol-6(su1006)]*
**ABZ243**	*hlh-1(ts);smg-6;* MYO-3::GFP	*hlh-1(cc561);smg-6(ok1794);raIs5[myo-3p::GFP::myo-3+rol-6(su1006)]*
**ABZ244**	*hlh-1(ts);*MYO-3::GFP	*hlh-1(cc561); stEx30[myo-3p::GFP::myo-3 + rol-6(su1006)]*

Supplementary Table 1. Strains used in this study. Strains were outcrossed with our lab N2 stock at least four times. MYO-3::GFP strains, RW1596 and ABZ244, were used only in Figure 2H .

Primer	Primer sequence - Forward	Primer sequence -Reverse
PD4605	F- AAGTTGTGAAGCAAAGAACGTGTC	R- GGAGTTGTTCGTCGGTCATT
VC1305	F- GGAGCACAAATATCAGTCAGGC	R- CGTCATCTCGTGTCCTTCCA
18S	F- CCGTTCTTAGTTGGTGGAGTG	R- GCTAAACACCGCTTATCCCT
hlh-1	F- GTCACCGCAAATGACATCAC	R- GGTAGGTGCAGTTGGAGCAT
hsp-90	F- GGACCAGAAACCCAGACGATA	R- ACGAAGAGAAGAGCACGGAA
sti-1	F- TGCTGCCTACAAGCAAAAGG	R- TCCAACTTCGACGGCTTTCT
unc-23	F- TGGAAAGGGTGATGAGAAC	R- TGGGGAGGTTAGTGGAGAT
dnj-24	F- CGTCAAGTCGAGTGCACAT	R- TTGCGTTCTTTCCAGGTTC
hsp-12.2	F- ATGTCCGCTATCGAGGTGAC	R- CGACCTCGAACTTTTCCTTG
pph-5	F- TTTCTCGGCACCAAATTATTG	R- GCAATTTCGGATGTGGAAC
chn-1	F- AACTGAGCCCAAACGAAGTG	R- ATGGTAGAGCGCTTTGGAGA
fkb-6	F- AGATCGCGGTGATCAATTCT	R- AATCCGAGCGAATTGTGAAT
dnj-2	F- TGACGCCAAGCTTTATCTCAT	R- GTTTCAGCCAGCATTCTCGT
hsp-17	F- ATCGTCGTTTTCCACCATTC	R- ATTGTTTGATCGGCCCAGTA
smg-2	F- TGGCTATTGGATATTTGTCG	R- TTTCGAGAGAAAATCAAGCAA
smg-7	F- AACGAGGATTCGGAACCTGAAT	R-TTTATGTAGCATGAAAAGAAAAA AAACCAA

Supplementary Table 2. Primer used in this work. Primers for PD4605 and VC1305 were used for sequencing. Other primes were used for qPCR.
